#  Geographic Linkage and Variation in *Cryptosporidium hominis*

**DOI:** 10.3201/eid1403.071320

**Published:** 2008-03

**Authors:** Rachel M. Chalmers, Stephen J. Hadfield, Colin J. Jackson, Kristin Elwin, Lihua Xiao, Paul Hunter

**Affiliations:** * National Public Health Service Microbiology, Swansea, Wales, UK; † Swansea University, Swansea, Wales, UK; ‡ Centers for Disease Control and Prevention, Atlanta, Georgia, USA; § University of East Anglia, Norwich, UK

**Keywords:** Cryptosporidium hominis, subtyping, GP60, travel, dispatch

## Abstract

UK *Cryptosporidium hominis* isolates have previously shown slight PCR fragment length polymorphism at multiple loci. To further investigate transmission, we conducted a case–control study and sequenced the GP60 locus from 115 isolates. Nine subtypes were identified; IbA10G2 predominated. Having a non-IbA10G2 subtype was significantly linked to recent travel outside Europe.

## Abstract 2

Geographic Linkage and Variation in *Cryptosporidium hominis*

*Cryptosporidium hominis*, a human-adapted species of the protozoan parasite *Cryptosporidium*, causes ≈50% of the reported cryptosporidiosis cases in the United Kingdom each year (*1*). Risk factors for *C. hominis* have been identified as traveling abroad and changing diapers of children (*2*). However, studies using multilocus fragment typing of mini- and microsatellite DNA markers have shown that *C. hominis* isolates from the United Kingdom are genetically very similar (*3*,*4*), and no associations between *C. hominis* subtype and risk have been identified (*4*). To explore whether a more detailed examination of genomic DNA could benefit public health, we used sequence analysis of the widely studied and highly variable GP60 gene to reexamine *C. hominis* isolates from a case–control study of sporadic cryptosporidiosis (*2*).

## The Study

A total of 115 *C. hominis* isolates were collected and confirmed during a case–control study of human cryptosporidiosis in Wales and northwest England (*2*). To identify subtypes, we analyzed the DNA sequences of an ≈850-bp region of the GP60 gene encompassing the polyserine tract (variable numbers and forms of a repeating sequence of 3 nucleotides coding for the amino acid serine) and the hypervariable downstream region (*5*). We used a nested PCR protocol with primary PCR primers AL3531 and AL3535 and secondary primers AL3532 and AL3534. PCR products were sequenced in both directions.

The microsatellite triplet codons were categorized according to the number of trinucleotide repeats (TCA, TCG, or TCT) coding for the amino acid serine (*6*), and the nomenclature was expanded for subtype family Ia to include the number of repeats (e.g., R1, R2) of the sequence AA(A/G)ACGGTGGTAAGG after the microsatellite region (*7*). Sequence data for representative isolates were deposited in GenBank (accession nos. EU161648–EU161655, EF214734, and EF214735). We then investigated subtypes for relationships with reported exposures by using single-variable analysis performed in SPSS 12.0 version (SPSS Inc., Chicago, IL, USA).

Of 115 *C. hominis* isolates, 14 were not typeable at the GP60 locus (12 did not amplify and 2 gave equivocal reactions); typeability was 87.8%. Nine subtypes were identified but 92 (91.1%) typeable isolates were IbA10G2. Each of the other identified types contained only 1 isolate member except for IgA24, which contained 2. This resulted in a low discriminatory power of 0.171.

More persons with subtypes other than IbA10G2 had a history of recent foreign travel (5/9, 55.6%) than did those with IbA10G2 (27/92, 29.3%), although this was not statistically significant (p = 0.1374 [Fisher exact test], odds ratio [OR] 3.01, 95% confidence interval [CI] 0.59–16.20). However, all 5 case-patients with other subtypes reported travel history outside Europe, 3 to Pakistan (subtypes IaA12R3, IaA22R2, and IaA30R3), 1 to Kenya (IaA25R3), and 1 to New Zealand (IgA24) while only 3 case-patients with IbA10G2 types were known to have traveled outside Europe (to Tunisia and Turkey) (Table 1). All those who reported travel within Europe had subtype IbA10G2.

**Table 1:** Travel history and *Cryptosporidium hominis* GP60 subtypes in sporadic cases, United Kingdom

Travel history	No. subtype IbA10G2	No. other subtypes	Other subtype details
No	65	4	IaA23R4, IbA9G2, IfA12G1, IgA24
Yes	27	5	See below
European travel destinations			
Balearic Islands	3	0	
Canary Islands	5	0	
Cyprus	3	0	
France	4	0	
Greece	2	0	
Spain	6	0	
Non-European destinations			
Kenya	0	1	IaA25R3
Pakistan	0	3	IaA12R3, IaA22R2, IaA30R3
New Zealand	0	1	IgA24
Tunisia	2	0	
Turkey	1	0	
Destination not known	1	0	

Four case-patients who had not traveled outside the United Kingdom had non-IbA10G2 alleles, but with the exception of IgA24, these were different from the subtypes found in case- patients who had traveled outside Europe. The relationship between travel outside Europe and GP60 subtypes was statistically significant (p = 0.00008 [Fisher exact test], OR 37.08, 95% CI 4.76–303.65; Table 2). No other epidemiologic associations were present.

**Table 2:** Travel outside Europe and *Cryptosporidium hominis* GP60  subtypes in sporadic cases, United Kingdom

Travel outside Europe	No. subtype IbA10G2	No. other subtypes	Total
No	88	4	92
Yes	3	5	8
Total	91	9	100

## Conclusions

Although GP60 sequence typing had very low discriminatory power for UK *C. hominis* isolates, our findings are in agreement with previous findings based on multiple loci that *C. hominis* appears to be highly conserved in the United Kingdom (*3*,*4*). DNA sequencing of a substantial proportion of the GP60 gene, including the microsatellite region, provides higher resolution data than investigating microsatellite length polymorphisms, which may mask differences in sequence (*8*); here, DNA sequencing facilitated identification of a significant link between subtype and foreign travel outside Europe. Subtype IbA10G2 is very clearly predominant in the United Kingdom. Subtype family Ib and the IbA10G2 subtype have been reported in Europe both in sporadic cases and outbreaks (*9*–*12*) and occur worldwide (*12*). The conclusion of Cohen et al. (*11*), that Ib is the predominant *C. hominis* allele associated with waterborne outbreaks, is explained if this is the most common allele causing human cryptosporidiosis in Europe, as it is in the United Kingdom, and is therefore predominant in human sewage.

In nonindustrialized countries, a greater variety of *C. hominis* subtypes have been reported (*7*,*8*,*13*,*14*). Of the 3 isolates found in case-patients returning from Pakistan, IaA12R3 had been isolated from a patient from Nepal (GenBank accession no. AY167595); IaA22R2 and IaA30R3 had not been reported previously. Subtype IaA25R3 was found in a case-patient returning from Kenya and was homologous to a *C. hominis* reference strain (TU502) of Ugandan origin (GenBank accession no. XM_663000). Notably, of the 4 case-patients with non-IbA10G2 subtypes who did not report foreign travel, 1 had the IgA24 subtype, which matched an isolate from Northern Ireland (GenBank accession no. EF214734), and may well circulate in the United Kingdom; IaA23R4 was homologous to isolates from the United States (GenBank accession no. AF164504) and Canada (GenBank accession no. DQ192510); and IfA12G1 had been identified in Australia (*12*).

*C. hominis* is highly conserved in indigenous UK case-patients, and subtypes other than IbA10G2 are linked to recent foreign travel outside Europe. It is not possible to predict whether this apparent stability will remain or whether it will be influenced by international travel.
